# *De novo* transcriptomic analysis of the female and male adults of the blood fluke *Schistosoma turkestanicum*

**DOI:** 10.1186/s13071-016-1436-2

**Published:** 2016-03-11

**Authors:** Guo-Hua Liu, Min-Jun Xu, Qiao-Cheng Chang, Jun-Feng Gao, Chun-Ren Wang, Xing-Quan Zhu

**Affiliations:** State Key Laboratory of Veterinary Etiological Biology, Key Laboratory of Veterinary Parasitology of Gansu Province, Lanzhou Veterinary Research Institute, Chinese Academy of Agricultural Sciences, Lanzhou, Gansu Province 730046 PR China; College of Animal Science and Veterinary Medicine, Heilongjiang Bayi Agricultural University, Daqing, Heilongjiang Province 163319 PR China; College of Animal Science, South China Agricultural University, Guangzhou, Guangdong Province 510642 PR China; Department of Parasitology, Heilongjiang Institute of Veterinary Science, Qiqihar, Heilongjiang Province 161006 PR China; Jiangsu Co-innovation Center for the Prevention and Control of Important Animal Infectious Diseases and Zoonoses, Yangzhou University College of Veterinary Medicine, Yangzhou, Jiangsu Province 225009 PR China

**Keywords:** *Schistosoma turkestanicum*, Transcriptome, Differentially expressed genes, Next-generation sequencing

## Abstract

**Background:**

*Schistosoma turkestanicum* is a parasite of considerable veterinary importance as an agent of animal schistosomiasis in many countries, including China. The *S. turkestanicum* cercariae can also infect humans, causing cercarial dermatitis in many countries and regions of the world. In spite of its significance as a pathogen of animals and humans, there is little transcriptomic and genomic data in the public databases.

**Methods:**

Herein, we performed the transcriptome Illumina RNA sequencing (RNA-seq) of adult males and females of *S. turkestanicum* and *de novo* transcriptome assembly.

**Results:**

Approximately 81.1 (female) and 80.5 (male) million high-quality clean reads were obtained and then 29,526 (female) and 41,346 (male) unigenes were assembled. A total of 34,624 unigenes were produced from *S. turkestanicum* females and males, with an average length of 878 nucleotides (nt) and N50 of 1480 nt. Of these unigenes, 25,158 (72.7 %) were annotated by blast searches against the NCBI non-redundant protein database. Among these, 21,995 (63.5 %), 22,189 (64.1 %) and 13,754 (39.7 %) of the unigenes had significant similarity in the NCBI non-redundant protein (NR), non-redundant nucleotide (NT) and Swiss-Prot databases, respectively. In addition, 3150 unigenes were identified to be expressed specifically in females and 1014 unigenes were identified to be expressed specifically in males. Interestingly, several pathways associated with gonadal development and sex maintenance were found, including the Wnt signaling pathway (103; 2 %) and progesterone-mediated oocyte maturation (77; 1.5 %).

**Conclusions:**

The present study characterized and compared the transcriptomes of adult female and male blood fluke, *S. turkestanicum*. These results will not only serve as valuable resources for future functional genomics studies to understand the molecular aspects of *S. turkestanicum*, but also will provide essential information for ongoing whole genome sequencing efforts on this pathogenic blood fluke.

## Background

Schistosomiasis is a neglected tropical disease caused by blood flukes (genus *Schistosoma*) that ranks second only to malaria among the parasitic diseases and affects more than 200 million people in a number of tropical and subtropical countries [1]. It is estimated that schistosomiasis causes an annual loss of between 1.7 and 4.5 million disability adjusted life years (DALYs) and contributes to several hundreds of thousands of deaths annually [2–4]. To date, no vaccines are available, and treatment relies on one drug, praziquantel [5].

*S. turkestanicum* (syn. *Orientobilharzia turkestanicum*) is a parasite of major veterinary importance as an agent of animal schistosomiasis, which infects a range of animals including cattle, sheep, goats, water buffaloes, horses, donkeys, mules, camels, and causes considerable economic losses [6]. Importantly, the cercariae of *S. turkestanicum* can also infect humans, in which they can cause cercarial dermatitis, and is considered the major pathogen of cercarial dermatitis in many countries and regions of the world, including China [6, 7]. In spite of its medical and veterinary importance, the genetics, epidemiology and biology of this parasite remain poorly understood.

Recent developments in deep sequencing or next-generation sequencing (NGS) provide an opportunity for rapid and cost-effective generation of genome-scale data [8, 9]. NGS technology, such as Solexa/Illumina platforms, has dramatically improved the efficiency of gene discovery, and is particularly suited to transcriptomics of distinct animal and plant taxa [10–13]. To date, many trematode transcriptomes have been sequenced using the NGS technology, such as *Fascioloides magna* [14]; *Clonorchis sinensis* [15]; *Eurytrema pancreaticum* [16], *Schistosoma japonicum* [17], *Schistosoma mansoni* [18], *Schistosoma haematobium* [19], *Opisthorchis felineus* [20], *Paramphistomum cervi* [21] and *Fasciola hepatica* [22]. In spite of the availability of advances in sequencing technologies and bioinformatic methods, there is still a paucity of knowledge of the complete transcriptome for this zoonotic fluke *S. turkestanicum*. Herein, a NGS platform and powerful *de novo* short-read assembly was employed to uncover a global view of the transcriptomes of adult male and female *S. turkestanicum* worms. The aim of the present study was to produce transcriptomic data to aid the better understanding of the biology of *S. turkestanicum*, which would facilitate the identification of intervention targets for *S. turkestanicum* and other medically and veterinary important trematodes.

## Methods

### Ethics statement

This study was approved by the Animal Ethics Committee of Lanzhou Veterinary Research Institute, Chinese Academy of Agricultural Sciences (Permit code. LVRIAEC2013-006). The sheep, from which *S. turkestanicum* was collected, were handled in accordance with good animal practices required by the Animal Ethics Procedures and Guidelines of the People’s Republic of China.

### Sample preparation

Adult blood flukes representing female and male *S. turkestanicum* (codes: OutF and OutM) were obtained from the portal and mesenteric veins of naturally infected sheep at a local slaughter house in Heilongjiang province, China. One blood fluke was collected from each of the five sheep, which were randomly selected for slaughter and sale, following the perfusion of the mesenteric and intestinal vessels using physiological saline (37 °C). Five blood flukes (three females and two males, respectively) were washed in physiological saline six times to remove any contamination with bacterial and host DNA, identified morphologically as *S. turkestanicum* according to existing keys and descriptions [23], and their identity was further ascertained by direct amplification and sequencing of the internal transcribed spacer (ITS) region, as previously described [24]. The male and female *S. turkestanicum* worms were immediately frozen in liquid nitrogen and stored at −80 °C until use.

### RNA isolation and Illumina sequencing

Total RNA was extracted from pooled females (*n* = 3) and pooled males (*n* = 2) of *S. turkestanicum,* respectively*,* using Trizol reagent (Invitrogen, Life Technologies, Carlsbad, CA, USA) according to the manufacturer’s protocol, and was stored at −80 °C until use. The Oligo (dT) was used to isolate poly (A) mRNA from total RNA. Mixed with the fragmentation buffer, the mRNA was fragmented into short fragments. Then cDNA was synthesized using the mRNA fragments as templates. Short fragments were purified and resolved with EB buffer for end reparation and single nucleotide A (adenine) addition. After that, the short fragments were connected with adapters. The suitable fragments were selected as templates for the PCR amplification. During the Quality Control (QC) steps, an Agilent 2100 Bioanaylzer and an ABI StepOnePlus Real-Time PCR System were used in quantification and qualification of the sample library. Illumina HiSeq™ 2000 was applied for sequencing at BGI-Shenzhen, Shenzhen, China according to the manufacturer’s instructions (Illumina, San Diego, CA, USA).

### *De novo* assembly

Prior to assembly, the high-quality clean reads were obtained from raw reads by removing adaptor sequences, highly redundant sequences, reads that contained more than 10 % ‘N’ rate (the ‘N’ character representing ambiguous bases in reads), and low quality reads containing more than 50 % bases with Quality (Q) value (Q-value ≤ 10). *De novo* assembly of the clean reads was performed by using the Trinity software, which was designed specifically for transcriptome assembly [25]. Briefly, Trinity (this is in Trinity software) first combines reads of a certain length of overlap to form longer fragments, which are called contigs. Then the reads are mapped back to contigs. Trinity connects the contigs and identifies sequences that cannot be extended on either end. Such sequences are defined as unigenes. Unigenes from each sample’s assembly can be used in further processes of sequence splicing and redundancy removal with sequence clustering software TGICL [26] in order to acquire non-redundant unigenes that are as long as possible.

### Bioinformatics analysis

Unigene sequences were first aligned to the NCBI non-redundant proteins (NR), non-redundant nucleotide (NT), Swiss-Prot, Cluster of Orthologous Groups (COG) and Kyoto Encyclopedia of Genes and Genomes (KEGG) databases by BLASTx, using an e-value <0.00001. The unigenes were tentatively annotated according to the known sequences with the highest sequence similarity. The annotated unigenes direction and predicted protein coding sequences (CDS) were identified by the best alignment results. ESTScan [27] was used to predict the CDS and the sequence direction when unigenes were unaligned to any of the databases. With NR annotation, the Blast2GO program was used to classify unigenes to Gene Ontology (GO) terms such as molecular function, biological processes, and cellular components [28]. After obtaining GO annotations for all unigenes, WEGO software [29] was used to perform GO function classification for all unigenes and to analyse the distribution of *S. turkestanicum* gene functions at the macro-level. Simple sequence repeats (SSRs) in the nucleotide sequences were identified using the MIcroSAtellite (MISA) identification tool [30]. The poly-A and poly-T sequences at the terminal regions of the UTs were removed before SSR identification. SOAPsnp [31] was used (with parameters -u t -Q i -L 90) on pileup files to output lists of single nucleotide polymorphisms (SNPs) and their locations.

### Identification of female and male differentially expressed genes

The calculation of Unigene expression used the FPKM method (Fragments Per kb per Million reads) [32]. RPKM was directly used to compare the difference of gene expression levels between females and males. False Discovery Rate (FDR) control is a statistical method used in multiple hypothesis testing to correct for *p*-value. In our analysis, we chose those with FDR ≤ 1 × 10^−3^ and |log_2_ ratio| ≥2 to identify sex-biased genes. Differentially expressed genes were then subjected to GO functional analysis and KEGG Pathway analysis.

## Results

### Sequencing and assembly

**Fig. 1:**
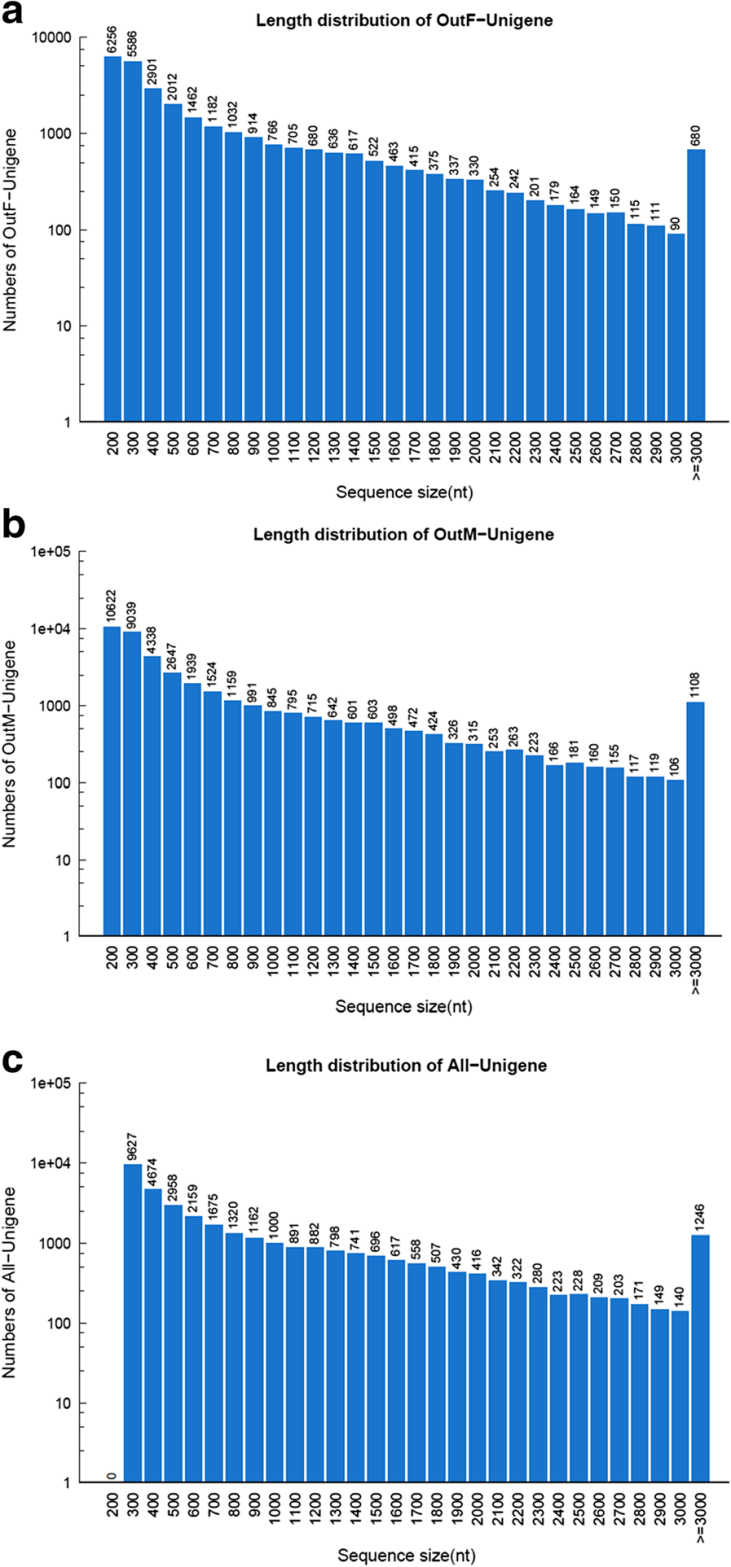
Length distribution of unigenes of adult *Schistosoma turkestanicum* transcriptome. **a**: Female- unigene; **b**: Male- unigene; **c**: All-unigene

Using Illumina (paired-end) sequencing technology, we obtained a total of 86,697,820 and 84,578,440 raw reads from female and male adults of *S. turkestanicum*, respectively. After quality filtration, 81,086,908 (total clean nucleotides: 7,297,821,720 nt) and 80,469,244 (total clean nucleotides: 7,242,231,960 nt) high quality clean reads were generated for assembly from females and males, respectively. All clean reads were submitted to the Sequence Read Archive database at NCBI (accession nos: SRP068812 and SRP068813). The Q20 percentage (Q20 percentage is the proportion of sequencing nucleotides error rate with quality value <1 %) and GC percentage of females were 97.69 % and 40.62 %, respectively. The Q20 and GC percentages of males were 97.86 % % and 41.26 %, respectively. Based on the clean reads, a total of 46,041 (>200 nt) and 69,543 (>200 nt) contiguous sequences (contigs) without gaps were produced by Trinity software for *S. turkestanicum* females and males, respectively (Table 1). Short unigenes (less than 200 nt) were removed. A total of 34,624 unigenes (total length: 30,412,123 nt) were produced from all samples (females and males), with an average length of 878 nt and N50 (A measure of the contig or scaffold length. It is the maximum length L such that 50 % of all nucleotides lie in contigs or scaffolds of size at least L) of 1480 nt. A total of 29,526 and 41,346 unigenes were produced by the clustering of *S. turkestanicum* sequences from females and males, respectively (Table 1). In females, 5356 unigenes (18.1 %) were longer than 500–1000 nt, and 3160 unigenes (10.7 %) were longer than 1000-1500 nt. In males, 6458 unigenes (15.6 %) were longer than 500–1,000 nt, and 3356 unigenes (8.1 %) were longer than 1000-1500 nt. All unigenes showed no gaps, thus demonstrating the high quality of Trinity assembly. The length distribution of these unigenes is shown in Fig. 1.

**Table 1:** Summary of transcriptome data for adult *Schistosoma turkestanicum*

Data generation and filtering	Female	Male
Raw reads	86,697,820	84,578,440
Clean reads	81,086,908	80,469,244
GC content	40.62 %	41.26 %
Q20 percentage	97.69 %	97.86 %
Contigs (mean length; N50)	46,041 (419 nt; 977 nt)	69,543 (355 nt; 699 nt)
Unigenes (mean length; N50)	29,526 (744 nt; 1363 nt)	41,346 (673 nt; 1324 nt)

### Functional annotation

In order to obtain and validate sequence-based annotations for all assembled unigenes, we employed Blastx for a sequence similarity search against the NR, NT, Swiss-Prot, GO, COG and KEGG databases, with an e-value <0.00001. Of these unigenes, 25,158 (72.7 %) were annotated by BLAST searches against the public databases. Among these, 21,995 (63.5 %), 22,189 (64.1 %), 13,754 (39.7 %), 11,816 (34.1 %), 5,889 (17 %) and 3,764 (10.9 %) unigenes had homologous sequences in NR, NT, Swiss-Prot, KEGG, COG and GO databases, respectively. Furthermore, 9,466 (27.34 %) unigenes showed no homology to known sequences deposited in these databases.

**Fig. 2:**
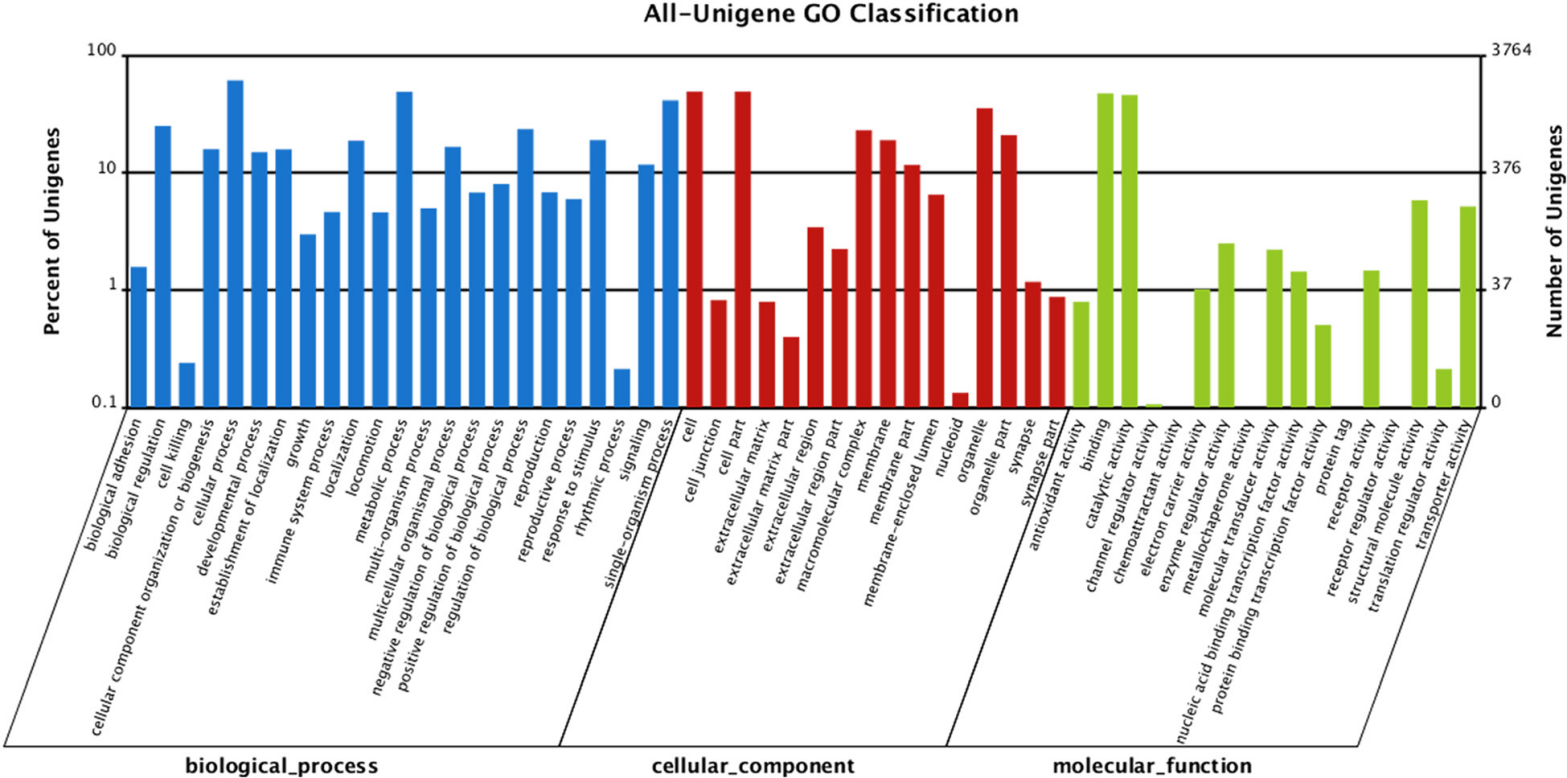
GO annotations of unigenes in *Schistosoma turkestanicum* transcriptome

A total of 3,764 unigenes were assigned 3,652 GO term annotations, which could be classified into three categories: biological process, molecular function, and cellular component. The biological process category consisted of 2,328 GO terms, which were assigned to 2,728 (7.9 %) unigenes. The cellular component category consisted of 495 GO terms, which were assigned to 2,108 (6.1 %) unigenes, and the molecular function category consisted of 829 GO terms, which were assigned to 2,965 (8.6 %) unigenes (Fig. 2). Within the biological process category, most unigenes (2,296) were assigned to “cellular processes”. In the cellular component category, most unigenes (1,844) were assigned to “cell”. In the molecular function category, the majority of unigenes (1,775) were associated with “binding” (Fig. 2).

**Fig. 3:**
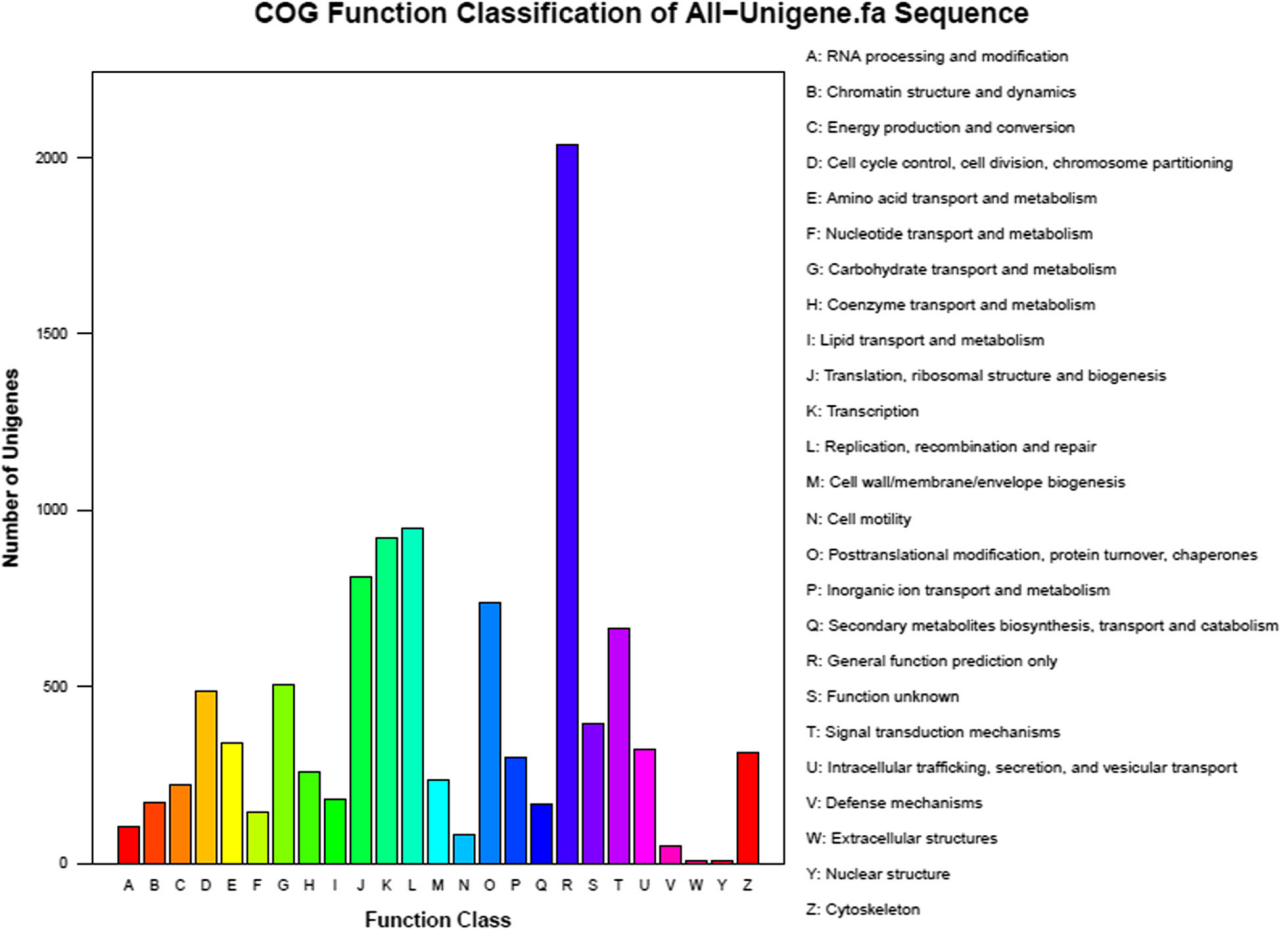
COG functional annotations of unigenes in *Schistosoma turkestanicum* transcriptome

All assembled unigenes were further annotated based on COG category. A total of 5,889 unigenes were further annotated, which could be grouped into 25 functional categories (Fig. 3). Among these functional categories, R (general function prediction only) included 5.9 % (2,036/34,624) unigenes, followed by L (replication, recombination and repair), K (transcription), J (translation, ribosomal structure and biogenesis), O (post-translational modification) and T (Signal transduction mechanisms) had 2.7 % (947/34,624), 2.7 % (923/34,624), 2.3 % (811/34,624), 2.1 % (739/34,624) and 1.9 % (666/34,624) unigenes, respectively. Furthermore, Y (Nuclear structure) had only six unigenes (Fig. 3).

Functional classification and pathway assignments were based on the KKEGG analysis. A total of 11,816 (34.1 %) uningenes were classified into six categories (Metabolism, Genetic information processing, Environmental information processing, Cellular processes, Organismal systems and Human disease) that mapped to 256 KEGG pathways. The top five KEGG pathways were metabolic pathways (1,545; 13.08 %), pathways in cancer (472; 3.99 %), regulation of actin cytoskeleton (444; 3.76 %), focal adhesion (443; 3.75 %) and spliceosome (401; 3.39 %) pathway. Interestingly, the ‘Immune system or Immune diseases’ sub-category, including 22 pathways, was associated with 1,802 (1,802/11,816; 15.3 %) KEGG annotated unigenes.

### SNP identification and microsatellite

**Fig. 4:**
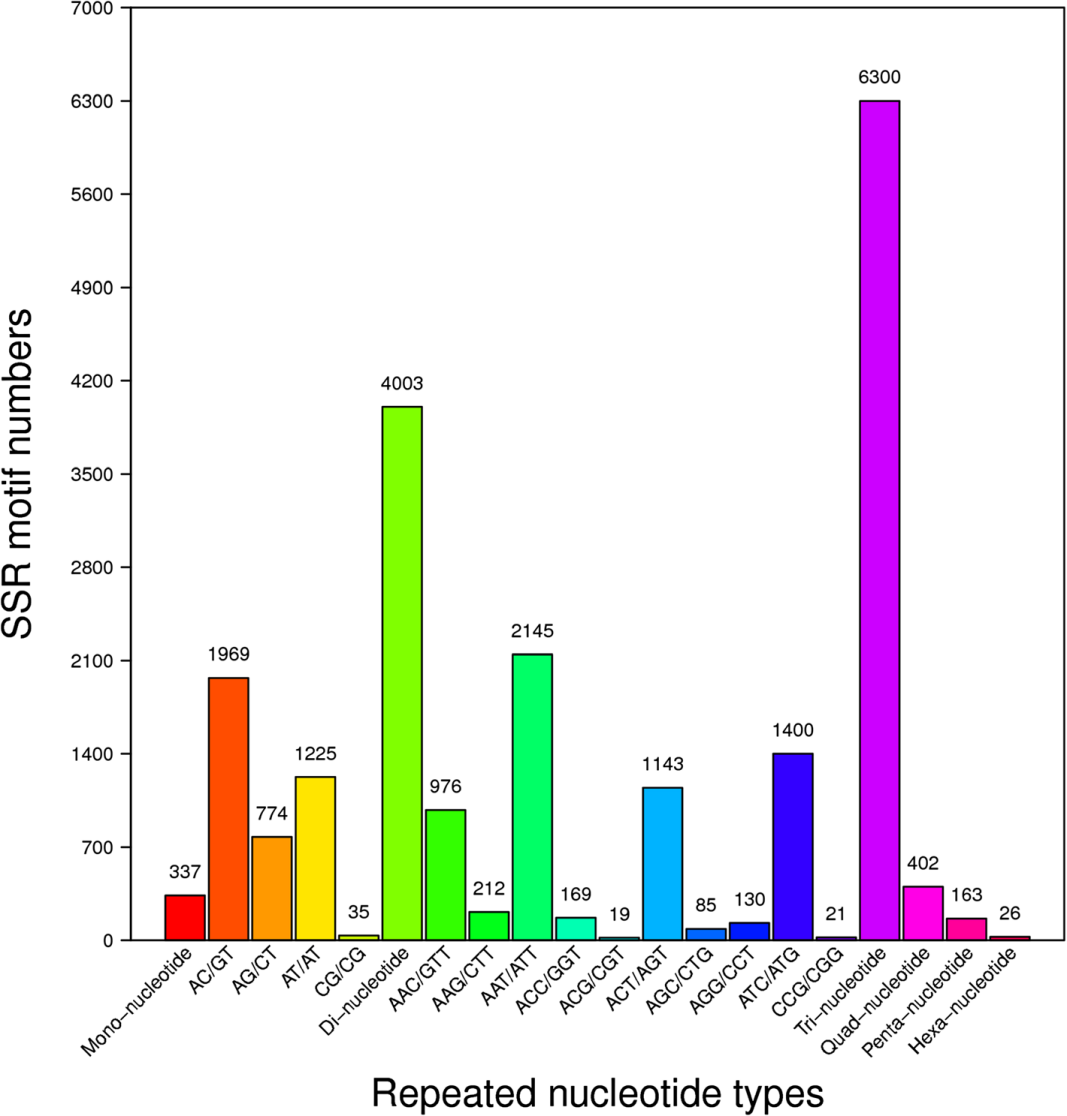
Repeated nucleotide type in *Schistosoma turkestanicum* transcriptome

In *S. turkestanicum* females, a total of 124,242 SNPs were also identified with the frequency of 1/58,738 nt, including transition 83,574 (A-G = 41,808; C-T = 41,766) and transversion 40,668 (A-C = 10,033; A-T = 14,360; C-G = 6,478; G-T = 9,797). In males, a total of 124,081 SNPs were also identified with the frequency of 1/58,366 nt, including transition 83,380 (A-G = 41,752; C-T = 41,628) and transversion 40,701 (A-C = 10,004; A-T = 14,264; C-G = 6,559; G-T = 9,874). Most of the repeats were repetitions of di-nucleotide or tri-nucleotide motifs. A total of 11,231 SSRs were identified with up to six nucleotide repeat lengths (Fig. 4).

### Unigene expression differences between *S. turkestanicum* females and males

**Fig. 5:**
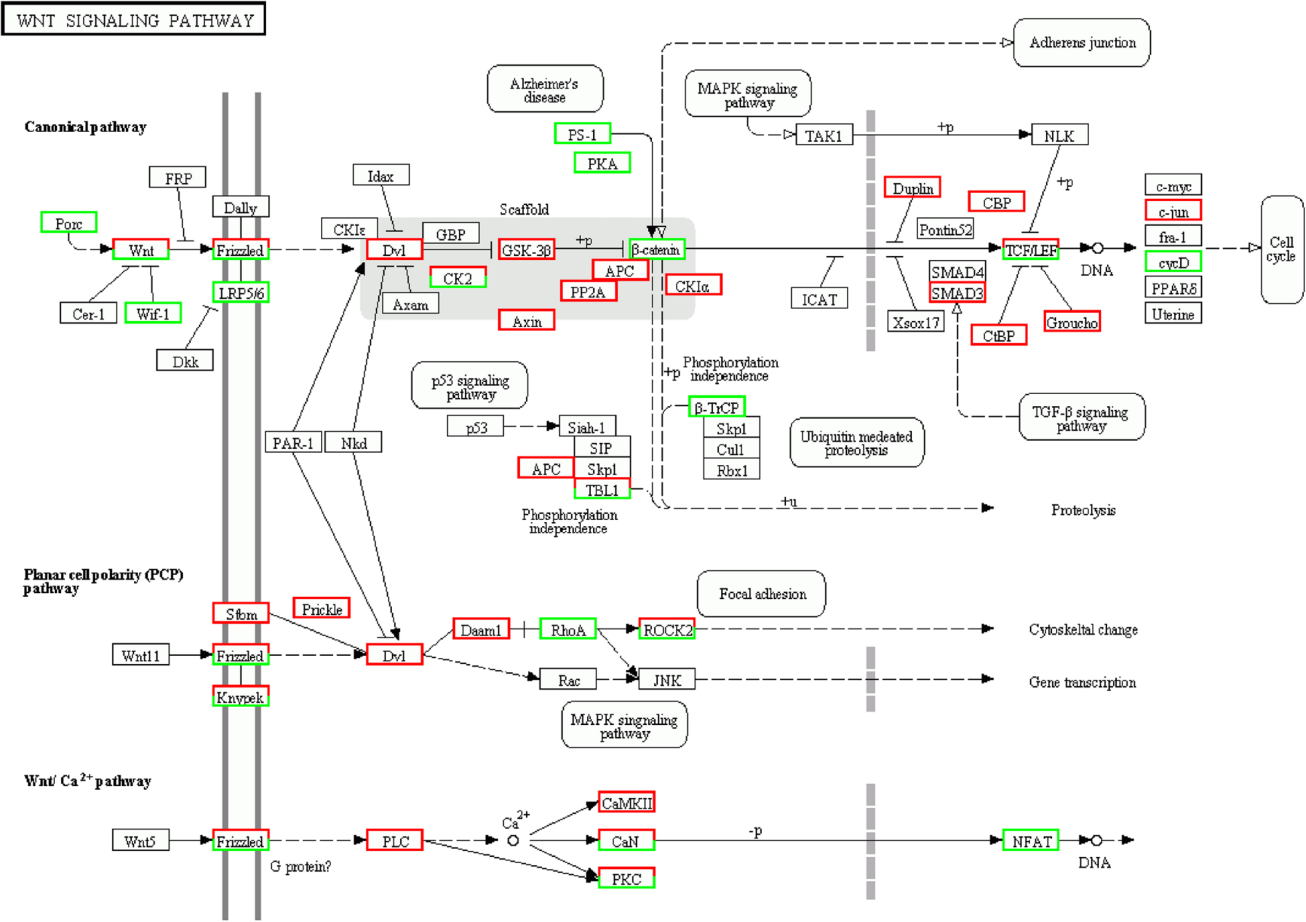
Wnt signaling pathway. Red background indicates up-regulated genes, green background indicates down-regulated genes

A total of 13,603 distinct genes were found differentially expressed between female and male worms, of which 8,021 genes were up-regulated and 5,582 genes were down-regulated in females *versus* males, respectively. In addition, 3,150 unigenes were identified to be expressed specifically in females and 1,014 unigenes were identified to be expressed specifically in males. The majority of sex-biased genes are in the metabolic pathways (663; 13 %) compared with other pathways. The significantly divergent pathways of differentially expressed genes mainly include pathways in cancer (234; 4.6 %), focal adhesion (220; 4.3 %), regulation of actin cytoskeleton (212; 4.2 %) and insulin signaling pathway (154; 3 %). Interestingly, several pathways associated with gonadal development and sex maintenance were found, such as the Wnt signaling pathway (103; 2 %), progesterone-mediated oocyte maturation (77; 1.5 %), GnRH signaling pathway (62; 1.2 %), TGF-beta signaling pathway (55; 1.1 %) and steroid hormone biosynthesis (12; 0.2 %) (Table 2 and Fig. 5).

**Table 2:** Key genes of the gonadal development and sex maintenance for adult females and males of *Schistosoma turkestanicum*

Pathway	Pathway ID	Representative genes
Wnt signaling pathway	ko04310	CL100.Contig1_All, CL100.Contig2_All, CL1218.Contig1_All, CL1218.Contig2_All, CL709.Contig2_All, CL785.Contig1_All, CL89.Contig1_All, Unigene10129_All, Unigene10266_All, Unigene10807_All, Unigene1085_All, Unigene11070_All, Unigene11334_All, Unigene1178_All, Unigene1358_All
Progesterone-mediated oocyte maturation	ko04914	CL1628.Contig1_All, CL1628.Contig2_All, CL174.Contig10_All, CL174.Contig5_All, CL174.Contig6_All, CL1781.Contig1_All, CL1781.Contig3_All, CL1963.Contig1_All, CL1963.Contig2_All, CL1963.Contig3_All
GnRH signaling pathway	ko04912	CL100.Contig1_All, CL100.Contig2_All, CL1536.Contig1_All, CL1536.Contig2_All, CL2320.Contig1_All, CL2423.Contig1_All, Unigene1009_All, Unigene10173_All, Unigene10497_All, Unigene10692_All, Unigene10930_All, Unigene10931_All, Unigene12392_All, Unigene12677_All
TGF-beta signaling pathway	ko04350	CL1563.Contig2_All, CL1604.Contig1_All, CL1989.Contig1_All, CL1989.Contig3_All, CL272.Contig1_All, CL272.Contig2_All, Unigene10807_All, Unigene1085_All, Unigene11334_All, Unigene1178_All, Unigene1318_All, Unigene15734_All, Unigene16098_All
Steroid hormone biosynthesis	ko00140	CL2632.Contig1_All, CL3396.Contig1_All, CL3396.Contig2_All, CL511.Contig1_All, CL511.Contig3_All, CL511.Contig4_All, CL688.Contig2_All, CL688.Contig3_All, Unigene11949_All, Unigene2063_All, Unigene6039_All, Unigene9729_All

## Discussion

Schistosomiasis caused by *Schistosoma* spp. remains a disease of considerable economic and medical importance in developing tropical and subtropical countries [33]. However, as a causative agent of human cercarial dermatitis and animal schistosomiasis, little is known of the transcriptome and genome of *S. turkestanicum*. Hence, this study decoded the trancriptomes of male and female *S. turkestanicum* by transcriptome sequencing.

Transcriptome sequencing is one of the most important tools for gene discovery. However, the traditional study method (large-scale EST sequencing) is time consuming and expensive. The NGS technology has become a powerful approach for high-throughput gene discovery on a genome-wide scale in organisms [9]. Due to its long read length, fast and accurate data, Solexa/Illumina have been the most widely used platforms for *de novo* transcriptome sequencing of many organisms, including some parasites [16, 34–37]. Recently, some studies have indicated that the relatively short reads can be effectively assembled with the great advantage of paired-end sequencing [38, 39], and the Illumina transcriptome *de novo* sequencing and assembly have been successfully used for distinct species [40–45]. Therefore, in the present study, a next-generation sequencing platform and powerful *de novo* short-read assembly method was employed to uncover a global view of the transcriptomes of female and male *S. turkestanicum* worms, and our results have also indicated that relatively short reads from Illumina paired-end sequencing can be effectively assembled.

In the present study, a great number of unigenes could match unique known proteins in public databases, which is similar with other trematodes [14–20]. A large number of unigenes were assigned to a wide range of GO categories and COG classifications, indicating that our data represented a wide diversity of transcripts. Based on the KEGG pathway, the top five KEGG pathways were metabolic pathways, pathways in cancer, regulation of actin cytoskeleton, focal adhesion and spliceosome pathway. These results indicated the active metabolic processes in *S. turkestanicum* development. Therefore, these results also strongly suggested that most of the genes involved in the different metabolic processes were identified through high-throughput Illumina transcriptome sequencing. Interestingly, the ‘Immune system or Immune diseases’ sub-category, including 22 pathways, was associated with 1,802 (1,802/11,816; 15.3 %) KEGG annotated unigenes, indicating that a much higher proportion of putative KO proteins of *S. turkestanicum* is involved in pathways associated with the immune system or immune diseases.

In the present study, a total of 13,603 distinct genes were found differentially expressed between *S. turkestanicum* females and males, which is significantly higher than that of *S. japonicum* [46], *S. mansoni* [18] and *S. haematobium* [19]. Interestingly, several pathways associated with gonadal development and sex maintenance were found, such as Wnt signaling pathway (103; 2 %). The Wnt signaling pathway is a conserved signaling network, which takes part in embryonic development, cell differentiation and proliferation, and the process of growth regulation. Wnt is a secreted glycoprotein, and many different Wnt gene subtypes have been found in a variety of animals. Wnt4 is regarded as a sex determination gene, which plays a key role in the morphological development of female mammals [47], and can regulate the formation of the Müllerian duct and the generation of ovarian steroids [48]. At birth, sexual development in males with a mutation in Wnt4 appears to be normal; however, Wnt4-mutant females are masculinized and the Müllerian duct is absent while the Wolffian duct continues to develop. Wnt4 may also be required for the maintenance of the female germ line [49]. Many genes in the Wnt signaling pathway were found in our transcriptomic database, such as casein kinase 1, epsilon, E1A/CREB-binding protein, serine/threonine-protein phosphatase 2A regulatory subunit A, WNT inhibitory factor 1 and F-box and WD-40 domain protein 1/11. Further research is required to elucidate the roles of these genes in the reproductive process of *S. turkestanicum*.

## Conclusions

The present study has produced the transcriptome data for adult female and male *S. turkestanicum* worms*,* and significantly enlarges the currently known gene repertoire of *S. turkestanicum*. These data will serve as a unique resource for future studies of *S. turkestanicum* gene functions, and will aid the ongoing whole genome sequencing efforts on this blood fluke. The characterization of these transcriptomic data has implications for the better understanding of the biology of *S. turkestanicum*, and will facilitate the development of intervention agents for this and other pathogenic flukes of human and animal health significance.

## Abbreviations

COG: cluster of orthologous groups
CDS: coding sequences
Contigs: contiguous sequences
DALYs: disability adjusted life years
FDR: false discovery rate
FPKM: fragments per kb per million reads
GO: gene ontology
ITS: internal transcribed spacer
KEGG: kyoto encyclopedia of genes and genomes
MISA: MIcroSAtellite
NGS: next-generation sequencing
NT: non-redundant nucleotide
NR: non-redundant protein
nt: nucleotides
QC: quality Control
RNA-seq: RNA sequencing
SSRs: simple sequence repeats
SNPs: single nucleotide polymorphisms

## References

[1] Xu J, Yu Q, Tchuenté LT, Bergquist R, Sacko M, Utzinger J (2016). Enhancing collaboration between China and African countries for schistosomiasis control. Lancet Infect Dis..

[2] King CH, Dickman K, Tisch DJ (2005). Reassessment of the cost of chronic helmintic infection: a meta-analysis of disability-related outcomes in endemic schistosomiasis. Lancet.

[3] Steinmann P, Keiser J, Bos R, Tanner M, Utzinger J (2006). Schistosomiasis and water resources development: systematic review, meta-analysis, and estimates of people at risk. Lancet Infect Dis.

[4] Makaula P, Sadalaki JR, Muula AS, Kayuni S, Jemu S, Bloch P (2014). Schistosomiasis in Malawi: a systematic review. Parasit Vectors.

[5] Doenhoff MJ, Cioli D, Utzinger J (2008). Praziquantel: mechanisms of action, resistance and new derivatives for schistosomiasis. Curr Opin Infect Dis.

[6] Wang CR, Chen J, Zhao JP, Chen AH, Zhai YQ, Li L (2009). *Orientobilharzia* species: neglected parasitic zoonotic agents. Acta Trop.

[7] Lawton SP, Majoros G (2013). A foreign invader or a reclusive native? DNA bar coding reveals a distinct European lineage of the zoonotic parasite *Schistosoma turkestanicum* (syn. *Orientobilharzia turkestanicum* (Dutt and Srivastava, 1955)). Infect Genet Evol.

[8] Knoppers BM, Zawati MH, Sénécal K (2015). Return of genetic testing results in the era of whole-genome sequencing. Nat Rev Genet.

[9] Mangiola S, Young ND, Korhonen P, Mondal A, Scheerlinck JP, Sternberg PW (2013). Getting the most out of parasitic helminth transcriptomes using HelmDB: implications for biology and biotechnology. Biotechnol Adv.

[10] Cárdenas-Conejo Y, Carballo-Uicab V, Lieberman M, Aguilar-Espinosa M, Comai L, Rivera-Madrid R (2015). *De novo* transcriptome sequencing in *Bixa orellana* to identify genes involved in methylerythritol phosphate, carotenoid and bixin biosynthesis. BMC Genomics.

[11] Liu J, Wang S, Qin T, Li N, Niu Y, Li D (2015). Whole transcriptome analysis of *Penicillium digitatum* strains treatmented with prochloraz reveals their drug-resistant mechanisms. BMC Genomics.

[12] Petrella V, Aceto S, Musacchia F, Colonna V, Robinson M, Benes V (2015). *De novo* assembly and sex-specific transcriptome profiling in the sand fly *Phlebotomus perniciosus* (Diptera, Phlebotominae), a major Old World vector of *Leishmania infantum*. BMC Genomics.

[13] Hagel JM, Morris JS, Lee EJ, Desgagné-Penix I, Bross CD, Chang L (2015). Transcriptome analysis of 20 taxonomically related benzylisoquinoline alkaloid-producing plants. BMC Plant Biol.

[14] Cantacessi C, Mulvenna J, Young ND, Kasny M, Horak P, Aziz A (2012). A deep exploration of the transcriptome and "excretory/secretory" proteome of adult *Fascioloides magna*. Mol Cell Proteomics.

[15] Huang Y, Chen W, Wang X, Liu H, Chen Y, Guo L (2013). The carcinogenic liver fluke, *Clonorchis sinensis*: new assembly, reannotation and analysis of the genome and characterization of tissue transcriptomes. PLoS One.

[16] Liu GH, Xu MJ, Song HQ, Wang CR, Zhu XQ (2016). *De novo* assembly and characterization of the transcriptome of the pancreatic fluke *Eurytrema pancreaticum* (Trematoda: Dicrocoeliidae) using Illumina paired-end sequencing. Gene.

[17] Wang X, Xu X, Lu X, Zhang Y, Pan W (2015). Transcriptome bioinformatical analysis of vertebrate stages of *Schistosoma japonicum* reveals alternative splicing events. PLoS One.

[18] Anderson L, Amaral MS, Beckedorff F, Silva LF, Dazzani B, Oliveira KC (2015). *Schistosoma mansoni* Egg, Adult male and female comparative gene expression analysis and identification of novel genes by RNA-Seq. PLoS Negl Trop Dis.

[19] Young ND, Jex AR, Li B, Liu S, Yang L, Xiong Z (2012). Whole-genome sequence of *Schistosoma haematobium*. Nat Genet.

[20] Pomaznoy MY, Logacheva MD, Young ND, Penin AA, Ershov NI, Katokhin AV (2015). Whole transcriptome profiling of adult and infective stages of the trematode *Opisthorchis felineus*. Parasitol Int.

[21] Choudhary V, Garg S, Chourasia R, Hasnani JJ, Patel PV, Shah TM (2015). Transcriptome analysis of the adult rumen fluke *Paramphistomum cervi* following next generation sequencing. Gene.

[22] Cwiklinski K, Dalton JP, Dufresne PJ, La Course J, Williams DJ, Hodgkinson J (2015). The *Fasciola hepatica* genome: gene duplication and polymorphism reveals adaptation to the host environment and the capacity for rapid evolution. Genome Biol.

[23] Dutt SC, Srivastava HD (1961). A revision of the genus *Ornithobilharzia * Odhner (1912), with the creation of two genera *Orientobilharzia* Dutt and Srivastava (1955) and *Sinobilharzia * Dutt and Srivastava (1955) (Trematoda: Schistosomatidae). Indian J Helminthol.

[24] Wang CR, Li L, Ni HB, Zhai YQ, Chen AH, Chen J (2009). *Orientobilharzia turkestanicum* is a member of *Schistosoma* genus based on phylogenetic analysis using ribosomal DNA sequences. Exp Parasitol.

[25] Grabherr MG, Haas BJ, Yassour M, Levin JZ, Thompson DA, Amit I (2011). Full-length transcriptome assembly from RNA-Seq data without a reference genome. Nat Biotechnol.

[26] Pertea G, Huang X, Liang F, Antonescu V, Sultana R, Karamycheva S (2003). TIGR Gene Indices clustering tools (TGICL): a software system for fast clustering of large EST datasets. Bioinformatics.

[27] Iseli C, Jongeneel CV, Bucher P. ESTScan: a program for detecting, evaluating, and reconstructing potential coding regions in EST sequences. Proc Int Conf Intell Syst Mol Biol. 1999;138–48. 10786296

[28] Conesa A, Gotz S, Garcia-Gomez JM, Terol J, Talon M, Robles M (2005). Blast2GO: a universal tool for annotation, visualization and analysis in functional genomics research. Bioinformatics.

[29] Ye J, Fang L, Zheng HK, Zhang Y, Chen J, Zhang ZJ (2006). WEGO: a web tool for plotting GO annotations. Nucleic Acids Res.

[30] Thiel T, Michalek W, Varshney R, Graner A (2003). Exploiting EST databases for the development and characterization of gene-derived SSR-markers in barley (*Hordeum vulgare* L.). Theor Appl Genet.

[31] Li R, Li Y, Fang X, Yang H, Wang J (2009). SNP detection for massively parallel whole-genome resequencing. Genome Res.

[32] Mortazavi A, Williams BA, McCue K, Schaeffer L, Wold B (2008). Mapping and quantifying mammalian transcriptomes by RNA-Seq. Nat Methods.

[33] Lai YS, Biedermann P, Ekpo UF, Garba A, Mathieu E, Midzi N (2015). Spatial distribution of schistosomiasis and treatment needs in sub-Saharan Africa: a systematic review and geostatistical analysis. Lancet Infect Dis.

[34] Sahoo PK, Kar B, Mohapatra A, Mohanty J (2013). *De novo* whole transcriptome analysis of the fish louse, *Argulus siamensis*: first molecular insights into characterization of Toll downstream signalling molecules of crustaceans. Exp Parasitol.

[35] Kumar M, Gantasala NP, Roychowdhury T, Thakur PK, Banakar P, Shukla RN (2014). *De novo* transcriptome sequencing and analysis of the cereal cyst nematode, *Heterodera avenae*. PLoS One.

[36] Wang F, Li D, Wang Z, Dong A, Liu L, Wang B (2014). Transcriptomic analysis of the rice white tip nematode, *Aphelenchoides besseyi* (Nematoda: Aphelenchoididae). PLoS One.

[37] Sun J, Wang SW, Li C, Hu W, Ren YJ, Wang JQ (2014). Transcriptome profilings of female *Schistosoma japonicum* reveal significant differential expression of genes after pairing. Parasitol Res.

[38] Maher CA, Palanisamy N, Brenner JC, Cao X, Kalyana-Sundaram S, Luo S (2009). Chimeric transcript discovery by paired-end transcriptome sequencing. Proc Natl Acad Sci U S A.

[39] Liu M, Qiao G, Jiang J, Yang H, Xie L, Xie J (2012). Transcriptome sequencing and de novo analysis for Ma bamboo (*Dendrocalamus latiflorus Munro*) using the Illumina platform. PLoS One.

[40] Wang Z, Fang B, Chen J, Zhang X, Luo Z, Huang L (2010). *De novo* assembly and characterization of root transcriptome using Illumina paired-end sequencing and development of cSSR markers in sweet potato (*Ipomoea batatas*). BMC Genomics.

[41] Kong F, Li H, Sun P, Zhou Y, Mao Y (2014). *De novo* assembly and characterization of the transcriptome of seagrass *Zostera marina* using Illumina paired-end sequencing. PLoS One.

[42] Liu S, Kuang H, Lai Z (2014). Transcriptome analysis by Illumina high-throughout paired-end sequencing reveals the complexity of differential gene expression during in vitro plantlet growth and flowering in *Amaranthus tricolor* L. PLoS One.

[43] Muriira NG, Xu W, Muchugi A, Xu J, Liu A (2015). *De novo* sequencing and assembly analysis of transcriptome in the Sodom apple (*Calotropis gigantea*). BMC Genomics.

[44] Sudheesh S, Sawbridge TI, Cogan NO, Kennedy P, Forster JW, Kaur S (2015). *De novo* assembly and characterisation of the field pea transcriptome using RNA-Seq. BMC Genomics.

[45] Liu SJ, Song SH, Wang WQ, Song SQ (2015). *De novo* assembly and characterization of germinating lettuce seed transcriptome using Illumina paired-end sequencing. Plant Physiol Biochem.

[46] Piao X, Cai P, Liu S, Hou N, Hao L, Yang F (2011). Global expression analysis revealed novel gender-specific gene expression features in the blood fluke parasite *Schistosoma japonicum*. PLoS One.

[47] Jordan BK, Mohammed M, Ching ST, Délot E, Chen XN, Dewing P (2001). Up-regulation of WNT-4 signaling and dosage-sensitive sex reversal in humans. Am J Hum Genet.

[48] Biason-Lauber A, Konrad D, Navratil F, Schoenle EJ (2004). A WNT4 mutation associated with Müllerian-duct regression and virilization in a 46,XX woman. N Engl J Med.

[49] Vainio S, Heikkilä M, Kispert A, Chin N, McMahon AP (1999). Female development in mammals is regulated by Wnt-4 signalling. Nature.

